# ASSOCIATION BETWEEN PERCEPTION OF SLEEP QUALITY AND ASSIMILATION OF CONTENT COVERED IN CLASS

**DOI:** 10.1590/1984-0462/;2018;36;3;00008

**Published:** 2018

**Authors:** Gabriel de Amorim Batista, Thaís Nadiane da Silva, Matheus Rodrigo de Oliveira, Paula Rejane Beserra Diniz, Samantha Sousa Lopes, Luciano Machado Ferreira Tenório de Oliveira

**Affiliations:** a Centro Universitário Tabosa de Almeida, Caruaru, PE, Brasil.; b Grupo de Pesquisa em Saúde e Esporte, Caruaru, PE, Brasil.; c Universidade de Federal de Pernambuco, Recife, PE, Brasil.

**Keywords:** Sleep, Learning, Adolescent, Students, Sono, Aprendizagem, Adolescente, Estudantes

## Abstract

**Objective:**

To analyze the association between self-perception of sleep quality and assimilation of content covered in classes by adolescents.

**Methods:**

Epidemiological cross-sectional study conducted with 481 high-school students (14 to 19 years), both genders, enrolled in public schools in the city of Caruaru, Pernambuco, and selected by random cluster sampling strategy. Analyses were conducted using the Chi-square test and binary logistic regression.

**Results:**

44.1% of the adolescents reported learning difficulties during classes, 77.1% slept less than eight hours per day, and 28.9% had a bad perception of their sleep quality. Young people who studied at least one extra hour per day out of school had less difficulty in assimilating class content (OR=0.34; 95%CI 0.19-0.58). We also found that participants who reported a bad perception of sleep quality were more likely to have learning difficulties at school (OR=1.73; 95%CI 1.13-2.65) regardless of gender, age, school shift, study time out of school, and sleeping hours.

**Conclusions:**

Perception of sleep quality was associated with learning difficulties at school regardless of the number of sleeping and study hours.

## RESUMO

**Objetivo:**

Analisar a associação, em adolescentes, entre a percepção da qualidade do sono e a assimilaçãodo conteúdo abordado em salas de aula.

**Métodos:**

Estudo epidemiológico, transversal, com abordagem quantitativa e abrangência municipal. A amostra foi selecionada por intermédio de uma estratégia de amostragem aleatória de cluster, sendo constituída por 481 estudantes (de 14 a 19 anos), de ambos os sexos, de escolas da rede pública estadual de ensino médio da cidade de Caruaru, Pernambuco. As análises foram realizadas por meio do teste do qui-quadrado e da regressão logística binária.

**Resultados:**

44,1% relataram dificuldade de assimilação do conteúdo abordado em sala, 77,1% dormiam menos de 8 horas e 28,9% tinham uma percepção ruim da qualidade do sono. Constatou-se que os jovens que estudavam pelo menos 1 hora por dia extraclasse tinham menos chance de ter dificuldade de assimilação do conteúdo abordado em sala de aula (OR=0,34; IC95% 0,19-0,58). Verificou-se que aqueles jovens que relataram ter uma percepção ruim da qualidade de sono tinham mais chances de ter dificuldade de assimilação do assunto abordado em sala (OR=1,73; IC95% 1,13-2,65), independentemente de sexo, idade, turno, tempo de estudo fora da sala de aula e quantidade de horas dormidas.

**Conclusões:**

A percepção da qualidade do sono, independentemente da quantidade de horas dormidas e do tempo de estudo extraclasse, foi associada com a dificuldade de assimilação do conteúdo abordado em sala de aula.

## INTRODUCTION

Sleep - a natural and vital process for the maintenance of body homeostasis - is of major importance for both body and brain to revigorate^1^ and is characterized by two stages. The first, Non-REM (non-rapid eye movement), encompasses: stage N, or sleep-wake transition; stage N2, period of greater stimulation of the parasympathetic nervous system; and stage N3, associated with slow-wave sleep (SWS)^2^ and release of growth hormone.^3^ ^,^ ^4^ Studies suggest that SWS plays a role in declarative memory consolidation.^5^ ^,^ ^6^ The second stage, known as rapid eye movement (REM), helps in synaptic remodeling and procedural memory processes.^2^ ^,^ ^5^ In this vein, we underline the importance of sleep in the process of memory consolidation and, consequently, learning.^5,6^

Nowadays, several elements may contribute to sleep deprivation in adolescents, including school pressure, socioeconomic features, excessive computer and cellular use, and pathological factors characterizing sleep disorders.^7^ ^,^ ^8^ Recent studies indicated that physical and mental exhaustion occurs during the wakefulness period after sleep deprivation, causing mood alterations, increased cortisol levels, and decreased attention levels, therefore influencing negatively the consolidation and recovery of daily information.^9^ ^,^ ^10^ Bearing in mind that sleep deprivation has consequences for school performance, Pereira et al.^11^ state that, to avoid daytime sleepiness, one must sleep at least eight hours and 33 minutes daily in school time.^11^

However, the ideal sleeping hours is a complex variable, for the literature describes two different sleeping profiles: short sleep, for people who require less than six hours of sleep per day; and long sleep, for those who need at least nine hours to feel revitalized.^12^ Furthermore, factors such as gender, age, and region of residence may influence daily sleeping time.^13^ Conversely, recent research points out that quality rather than amount of sleep would be associated with better school performance.^14^

Another factor probably related to the assimilation of content addressed in class is chronotype (morning and afternoon), which directly affects the adaptation of an individual to an environment, compromising their attention during class.^15^ Although the benefits of sleep quality are well-known,^16^ the literature lacks studies assessing its link with learning. In addition, there is no record of research taking into account the time of study out of school, an important control variable, since this habit may result in greater uptake and assimilation of information addressed at school.^17^ That being said, the objective of this study was to conduct an analysis on the association between self-perception of sleep quality and adolescents’ assimilation of content addressed in class, adjusting for possible confounding variables such as extra class study time and sleeping hours.

## METHOD

This is a descriptive study with quantitative approach, part of a cross-sectional epidemiological survey. The sample was composed of 481 students aged 14 to 19 years, both sexes, regularly enrolled in one of the 15 schools of the State school network in the city of Caruaru, Pernambuco, selected from a total of 8,833 institutions based on data from the Education and Culture State Secretariat.

The study was approved by the Human Research Ethics Committee of *Centro Universitário Tabosa de Almeida - Asces/Unita* (CAAE-22210913.8.0000.5203/CEP-ASCES: 072403/2013). Subjects’ participation was voluntary and anonymous, with an informed acceptance form being adopted for participants under 18 years of age and the informed consent form for parents/caregivers. Subjects older than 18 only signed the informed consent form.

To calculate the sample size, the following parameters were adopted: 95% confidence interval (95%CI); statistical power of 80%; maximum tolerable risk of error of 2 percentage points; drawing effect (deff)=2; and, because this is a study covering the analysis of multiple risk behaviors and with different frequency of occurrence, prevalence was estimated at 50%. Additionally, in order to lighten the limitations imposed by possible application losses and/or inadequate completion of questionnaires, the sample size was increased by 20%.

To select the required sample, a two-stage cluster sampling procedure was used: “period of the day” and “class” represented the sample units in the first and second stages, respectively. All schools in the public network of Caruaru were considered eligible for the study. In the first stage, the study period (daytime or nighttime) was adopted as stratification criterion. In the second stage, classes of the first, second and third terms were considered. From classes drawn, all students, regardless of age, were invited to participate in the study. After application of questionnaires, answers by students older than the established age (19 years) were excluded.

The questionnaires were applied in classrooms, in the form of a press conference without the presence of high-school teachers. However, the students were continuously assisted by researchers (two professors and three undergraduate students) to clarify any questions while completing the questionnaires. Personal information, socioeconomic and sociodemographic variables were obtained through the translated and adapted version of the Global School-based Student Health Survey (GSHS), proposed by the World Health Organization (WHO),^18^ which has been previously validated and is commonly used in studies involving adolescents.^19^ ^,^ ^20^

Self-perception of sleep quality was measured by the question: “How do you rate the quality of your sleep?”, the answers being dichotomized into “Sleep well” (for those who rated their sleep quality as “good”, “very good” and “excellent”) and “Do not sleep well” (for those who rated it as “bad” or “regular”). Self-perception of assimilation of content addressed in class was evaluated with the question: “Do you have difficulties assimilating the content addressed during classes?”, whose answers were dichotomized into “Yes” (for those who reported assimilation difficulties) and “No” (for those who did not report difficulties). The variable “sleeping hours” was measured by the question “How many hours do you sleep at night on average?”, with two choices of answers: “>8” and “<8”. Reproducibility indicators presented moderate to high intraclass correlation coefficient (0.62-1.00) for the variables used in this study (gender, age, study shift, time of study out of school, perception of sleep quality, sleeping hours, and difficulty assimilating content covered in classes).

Final data tabulation was done with the help of Epi-Data version 3.1 (Epidata Association, Odense, Denmark), a public-domain system with which electronic data input control procedures were also made through the function “check” (controls). In order to detect errors, the input of data was repeated and, through the function “duplicate files comparison”, the typing errors were detected and corrected.

The analyses were performed with the Statistical Package for the Social Sciences version 10.0 (SPSS Inc., Chicago, IL, USA). Upon descriptive analysis, the frequency distribution was observed. In inferential analysis, Pearson’s chi-square test was used to assess the association between perception of sleep quality and perception of content assimilation in class, besides the variables added to the model to explore possible confounding factors and to identify the need for statistical adjustment in analyses.

In the multivariate analysis, binary logistic regression was used, by estimating odds ratio and 95%CI to express the degree of association between independent variables (sleeping hours and perception of sleep quality) and dependent variable (perception of assimilation of content addressed in class), with the possible confounding variables controlled (hours of study out of school, perception of sleep quality, sleeping hours, and gender). Regarding confounding variables, they were all inputted simultaneously by the “Backward” method, only remaining in the statistical model those presenting p<0.20. After the predictive variables of the final model were obtained, occurrence of interaction was tested. For all tests, significance level was set at p<0.05.

## RESULTS

At the time of data collection, 569 students were present, but 31 did not agree to participate and 26 did not obtain parental consent, totaling 57 refusals. Thus, 512 students participated in the study, of which 481 were included because they were aged 14 to 19 years old. Participants were distributed among 9 of the 15 schools of the public network in the city of Caruaru, Pernambuco. From the total number of students who filled in the questionnaire, 54.1% were females. The characteristics of adolescents are shown in Table 1.

**Table 1:** Socioeconomic, demographic, and sleep characteristic in high school students in the public education network.

	Male		Female		Total		p-value^#^
	(n=221)		(n=260)		(n=481)		
	n	%	n	%	n	%	
Age (years)							
14-15	72	32.7	111	42.7	183	38.1	0.088
16-17	111	50.5	108	41.5	219	45.6	
18-19	37	16.8	41	15.8	78	16.3	
School shift							
Morning	63	28.5	67	25.8	130	27.0	0.192
Afternoon	36	16.3	34	13.1	70	14.6	
Night	42	19.0	40	15.4	82	17.0	
Full or part-time	80	36.2	119	45.8	199	41.4	
Years in school							
>8 years of education	25	13.6	22	9.9	47	11.6	0.249
<8 years of education	159	86.4	200	90.1	359	88.4	
Difficulty assimilating content addressed in classes							
No	132	59.7	137	52.7	269	55.9	0.121
Yes	89	40.3	123	47.3	212	44.1	
Sleeping hours							
>8 hours	43	19.5	67	25.8	110	22.9	0.100
<8 hours	178	80.5	193	74.2	371	77.1	
Perception of sleep quality							
Good	159	71.9	183	70.4	342	71.1	0.707
Bad	62	28.1	77	29.6	139	28.9	
Time of study out of school							
>1 hour	77	34.8	95	36.5	172	35.8	0.096
<1 hour	71	32.1	106	40.8	177	36.8	
Does not study out of school	73	33.1	59	22.7	132	27.4	

^#^Pearson’s chi-square test.

The prevalence of adolescents with difficulty in assimilating subject matters covered in classes, who slept less than eight hours per day, and had a poor perception of their sleep quality was 44.1%, 77.1% and 28.9%, respectively, with no significant differences between genders (p=0.121, p=0.100 and p=0.707, respectively).

**Figure 1:**
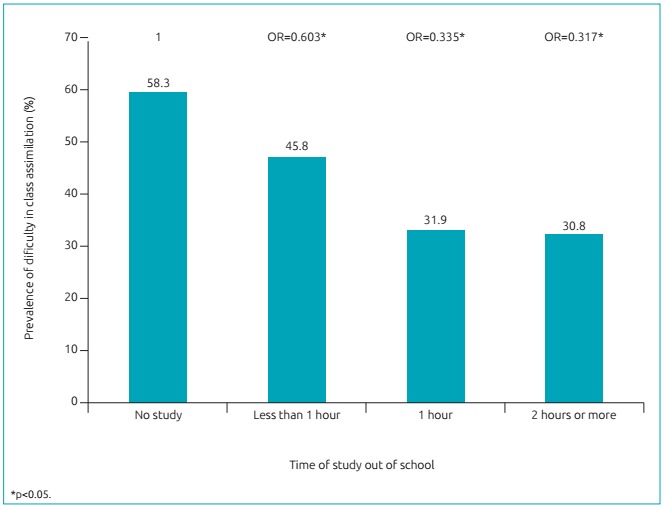
Prevalence of adolescents with difficulty in assimilating content addressed in class according to time of study out of school among high schoolers of the public education network of Caruaru, Pernambuco.

An inversely significant relation was found between hours of study out of school and difficulty assimilating content addressed in classes (Figure 1). Through logistic regression analysis, participants who studied more than one hour per day out of school were found to be less likely to have difficulty assimilating content addressed in classes (Figure 1), which proves that this is an important control variable.

Adolescents who rated their sleep quality as bad were more likely to have difficulties assimilating subjects addressed in class (OR=1.73; 95%CI 1.13-2.65) regardless of their sleeping hours, hours of study out of school, and gender. The variable sleeping hours was not associated with difficulty assimilating school content (p=0.492) (Table 2).

**Table 2:** Odds ratio for assimilation of content addressed in class, sleeping hours and perception of sleep quality among high schoolers of the public education network of Caruaru, Pernambuco.

	Difficulty in assimilating content addressed in class			
	Odds ratio	95%CI	Odds ratio	95%CI
	Crude		Adjusted	
Sleeping hours^#^				
>8 hours	1	0.61-1.43	1	0.536-1.35
< 8 hours	0.93		0.85	
Perception of sleep quality^†^				
Good	1	1.13-2.50	1	1.13-2.65
Bad	1.68*		1.73*	

95%CI: 95% confidence interval; #adjusted for gender, age, shift, time of study out of school, and perception of sleep quality; ^†^adjusted for gender, age, shift, time of study out of school, and total sleeping hours; *p<0.05.

## DISCUSSION

The purpose of this study was to investigate possible associations between self-perception of sleep quality and hours, and the perception of adolescents about assimilation of content addressed in class, controlling possible confounding variables such as gender and hours of extra class study.

In total, 44.1% of the adolescents reported difficulty in assimilating content addressed in class. participants who reported more than one extra hour of study out of school were less likely to have difficulty assimilating content. Self-perception of sleep quality rather than amount of sleep was associated with difficulty concentrating, regardless of sleeping hours, hours of extra class study, and gender.

A concerning finding of this study was that almost half of participants reported difficulty in assimilating school content, which is in line with results recently reported by Carneiro and Coutinho;^21^ the authors evaluated complaints related to schooling among cases taken care of at a mental health service and described 47% of the sample with learning difficulties. This deserves further detailed investigation so the meaning and origin of these difficulties are clarified.

School performance has been commonly evaluated in recent studies through student’s grades; ^14^ ^,^ ^16^ however, the grade itself would be the product a work performed. As reported by Dewald et al. in a meta-analysis,^22^ the most appropriate means to evaluate learning perception would be self-report by students, since other subjective methods overestimate outcomes. In this study, the difficulty in assimilating content addressed in class was evaluated through specific questions; therefore, results are based on individuals’ self-perception. In this sense, the importance of evaluating students throughout the learning process rather than the final result of this process (the grade) is emphasized so that adaptations are made to reach the expected outcomes.

Adolescents reporting at least one extra hour of study per day considerably minimizes the difficulty in content assimilation. According to Oliveira and Gastal,^17^ the application of different techniques and methodologies can serve as a strategy for better contextualization of knowledge, for both capturing new information and consolidating what has been already gained, besides placing the after-school environment as an important learning booster.

No significant association was found between amount of sleep and school content assimilation. Daily sleeping hours is an individual issue and these recommendations may vary according to external factors such as socioeconomic level, or internal factors such as sleeping profile - it is thus emphasized that the necessary sleeping hours for body and brain to reinvigorate may differ between individuals.^12^ ^,^ ^23^

The perception of sleep quality was associated with difficulty of content assimilation in class regardless of gender, age, study shift, extra study hours out of school, and sleeping hours. This finding may be related to the fact that poor sleep quality leads to increased fatigue, stress, and daytime sleepiness, ^24^ ^,^ ^25^ ^,^ ^26^ possibly making it difficult to assimilate content in class. Another factor worth mentioning is school stress, which could single-handedly influence content assimilation and learning negatively.^27^ Thus, information obtained during lessons will be naturally poorly understood and stored first in the hippocampus, which is responsible for short-term memory.^1^

During sleep, high activity occurs in the hippocampus to increase stimulation of cortical neurons and, afterwards, to pass the information on to the cerebral cortex,^28^ which will store the information in the long term.^29^ Aware that SWS helps consolidate memory while REM boosts procedural memory - and that both are responsible for consolidating information obtained -,^30^ we emphasize that good perception of sleep quality, associated with extra study hours, can reduce content assimilation difficulty, thus contributing to a more effective learning. That being put, schools should provide strategies to improve students’ learning, such as after-shift naps, instructions for a good night’s sleep, and information about their benefits with a view to more efficient learning.^31^

This study has some limitations that should be mentioned. The cross-sectional design prevents researchers from establishing a causal relationship between perception of sleep quality and difficulty assimilating content addressed in class. Sleep quality was self-reported; but even aware of the limitations related to the questionnaire, the reproducibility indicators showed moderate to high intraclass correlation coefficients for items used in the questionnaire. The representative sample can be pointed as one of the strengths of this study, since the sampling procedures were established to ensure that the population was composed of adolescent students attending schools in different shifts, in addition to the result being established after adjustment to potential confounding variables.

The results of this research highlight the need for a systematic diagnostic evaluation to verify the reasons related to difficulties in assimilating content addressed in class, so that specific interventions are carried out to minimize such problems and enhance students’ school performance. It should be emphasized that greater attention should be given to one’s self-perception of sleep quality and extra study hours among adolescents, aiming at a better assimilation of content covered at schools. Further research with a qualitative approach with participants who reported assimilation difficulties could be interesting, so that well-driven actions on specific reasons that lead to such difficulties can be carried out.

In conclusion, the perception of sleep quality, regardless of sleeping and extra study hours, was associated with difficulty in assimilating content, as perceived by students. Other points worth mentioning are: almost half of participants reported problems assimilating content approached in class, and those who reported at least one extra study hour a day also described less difficulty in class.
